# Export of Rgg Quorum Sensing Peptides is Mediated by the PptAB ABC Transporter in *Streptococcus Thermophilus* Strain LMD-9

**DOI:** 10.3390/genes11091096

**Published:** 2020-09-19

**Authors:** Abarna Lingeswaran, Coralie Metton, Céline Henry, Véronique Monnet, Vincent Juillard, Rozenn Gardan

**Affiliations:** Université Paris-Saclay, INRAE, AgroParisTech, Micalis Institute, 78350, Jouy-en-Josas, France; abarna.lingeswaran@inrae.fr (A.L.); coraliemetton@yahoo.fr (C.M.); celine.henry@inrae.fr (C.H.); veronique.monnet@inrae.fr (V.M.); vincent.juillard@inrae.fr (V.J.)

**Keywords:** signaling peptide, streptococci, RRNPP, transcriptional regulation

## Abstract

In streptococci, intracellular quorum sensing pathways are based on quorum-sensing systems that are responsible for peptide secretion, maturation, and reimport. These peptides then interact with Rgg or ComR transcriptional regulators in the Rap, Rgg, NprR, PlcR, and PrgX (RRNPP) family, whose members are found in Gram-positive bacteria. Short hydrophobic peptides (SHP) interact with Rgg whereas ComS peptides interact with ComR regulators. To date, in *Streptococcus thermophilus*, peptide secretion, maturation, and extracellular fate have received little attention, even though this species has several (at least five) genes encoding Rgg regulators and one encoding a ComR regulator. We studied pheromone export in this species, focusing our attention on PptAB, which is an exporter of signaling peptides previously identified in *Enterococcus faecalis*, pathogenic streptococci and *Staphylococcus aureus*. In the *S. thermophilus* strain LMD-9, we showed that PptAB controlled three regulation systems, two SHP/Rgg systems (SHP/Rgg_1358_ and SHP/Rgg_1299_), and the ComS/ComR system, while using transcriptional fusions and that PptAB helped to produce and export at least three different mature SHPs (SHP_1358_, SHP_1299_, and SHP_279_) peptides while using liquid chromatography-tandem mass spectrometry (LC-MS/MS). Using a deep sequencing approach (RNAseq), we showed that the exporter PptAB, the membrane protease Eep, and the oligopeptide importer Ami controlled the transcription of the genes that were located downstream from the five non-truncated *rgg* genes as well as few distal genes. This led us to propose that the five non-truncated *shp*/*rgg* loci were functional. Only three *shp* genes were expressed in our experimental condition. Thus, this transcriptome analysis also highlighted the complex interconnected network that exists between SHP/Rgg systems, where a few homologous signaling peptides likely interact with different regulators.

## Highlights

- PptAB exports signaling peptides from intracellular quorum sensing systems in *Streptococcus thermophilus*;
- PptAB only exports mature signaling peptides;
- in the *S. thermophilus* strain LMD-9, PptAB, Eep (a protease), and Ami (a peptide importer) are involved in a QS mechanism that controls the expression of genes located downstream all *rgg* genes but also of a few distal targets; and,
- at the strain level, crosstalk between the SHP/Rgg systems likely occurs and involves a few SHP signaling peptides that interact with multiple Rgg regulators.

## 1. Introduction

Quorum sensing (QS) is a cell-cell communication mechanism that is used by bacteria to trigger the expression of an entire set of genes when population levels exceed a certain threshold of cell density [1]. Functions that are controlled by QS are very diverse, ranging from luminescence in *Vibrio fischeri* [2], natural transformation in streptococci [3], bacteriocin production in lactococci and bacilli [4], and virulence in *Staphylococcus aureus* [5]. The QS circuit is similar across all bacteria. First, a signaling molecule, also called an autoinducer, is released into the extracellular environment, where it accumulates. Once it reaches a threshold concentration, its presence is detected by a sensor protein, which leads cells to modulate the expression of target genes. In Gram-positive bacteria, the signaling molecules are mainly autoinducing peptides (AIPs), also called pheromones. Most are ribosomally synthesized from short coding sequences (short CDSs), transformed into their mature forms, exported, and sometimes experience cyclization or the modification of amino acid residues. Some AIPs are produced via the proteolytic degradation of lipoproteins and, more specifically, their secretory signal peptides [6]. There are two general activation pathways. The first pathway is a two-component system that comprises a histidine kinase and a response regulator. The histidine kinase senses the presence of AIPs in the extracellular environment. A phosphorylation cascade involving the histidine kinase and the response regulator leads to the activation or repression of target gene transcription [5,7,8]. In the second pathway also called intracellular pathway, the presence of AIPs is sensed within the cell after they have been internalized by an oligopeptide permease transport system composed of ABC-family transporters. Once in the cell, AIPs interact with transcriptional regulators or Rap proteins, both members of the RRNPP family (which stands for Rap, Rgg, NprR, PlcR, and PrgX), thereby modifying the latter’s activity and, consequently, the expression of target genes [9,10]. Rap proteins mediate sporulation, competence, and the production of degradative enzymes in bacilli [11]. PlcR and NprR help to control virulence and necrotrophism in bacteria of the *cereus* group [12]. PrgX-like proteins are involved in plasmid transfer in enterococci [13]. The function of the Rgg-like regulators found in streptococci will be described below. To date, research focusing on this process has largely examined the interactions between RRNPP proteins and their cognate AIPs [10]. It has revealed that all of the members of the RRNPP family share a similar core structure and they have a tetratricopeptide domain in their C-terminus [14,15]. This domain is involved in the interaction with their cognate AIPs. In addition, the transcriptional regulators have a helix-turn-helix DNA-binding domain at their N-terminus. Interestingly, members of the RRNPP family each undergo distinct structural changes following binding with their specific AIPs [10].

Far less is known about the secretion, maturation, and extracellular fate of the AIPs, but these processes appear to be somewhat specific for each member of the RRNPP family. AIPs that are associated with Rap, PlcR, and NprR proteins have a particular secretion signal peptide in their N-terminus that is likely targeted for the Sec-dependent export pathway, resulting in the AIPs’ secretion and maturation via a signal peptidase. Additional extracellular proteases mediate further maturation: they have been identified for some AIPs of Rap proteins [11,16] and the AIP of the PlcR regulator [17]. Most, however, remain unknown. Peptides associated with PrgX-like regulators fall into one of two categories: (1) they are inhibitory peptides encoded by short genes or (2) they are activating peptides that are embedded in lipoprotein secretion signal peptides that are released outside the cell during lipoprotein export. Activating peptides are matured by serial peptidases, which include a lipoprotein signal peptidase, a transmembrane protease, named Eep, and additional undescribed exoproteases. Eep appears to be capable of maturing inhibitory peptides [18]. By screening a transposon mutant library, the researchers identified PptAB, a peptide exporter of ABC transporters, and determined that it was the exporter of three different activating peptides in *E. faecalis* [19]. The final members of the RRNPP family—Rgg and ComR—are Rgg-like regulators. Rgg regulators help to produce modified secreted peptides [20], biofilms and lysozyme resistance [21,22,23], and mediate pathogenicity [24,25]. ComR regulators help to mediate competence and bacteriocin production [3,26]. Rgg regulators are associated with AIPs, called short hydrophobic peptides (SHPs); ComR regulators are associated with ComS peptides, called *sigX*-inducing peptides (XIPs) in their mature form. Eep causes the maturation of SHPs, as observed for one SHP in *Streptococcus thermophilus* [27]; for SHP2 and SHP3 in *Streptococcus pyogenes* [21]; and, for the RovS-associated SHP in *Streptococcus agalactiae* [24]. Eep also controls the maturation of ComS in *S. thermophilus* [28], but only plays a contributing role in *S. mutans* [29]. Finally, PptAB is involved in the export of SHP2 and SHP3 in *S. pyogenes* [30] and of the RovS-associated SHP in *S. agalactiae* [24]; however, it only contributes to the export of mature ComS in *Streptococcus mutans* [30].

*S. thermophilus* plays a major role in the food industry, because it is a starter employed in massive quantities to manufacture yoghurt, Swiss-type cheeses, and Italian-type cheeses. It is also a useful representative of the genus *Streptococcus* with which to study QS: *S. thermophilus* encodes many QS systems in its genome, including at least five different SHP/Rgg systems [27] and one ComS/ComR system [31]. We have deciphered two different SHP/Rgg systems in the *S. thermophilus* wild-type strain LMD-9. The first system, SHP/Rgg_1358_, controls the transcription of the *shp_1358_* gene and at least three genes that are involved in the production of a cyclic secreted peptide, called streptide, which are located downstream from the *rgg*_1358_ gene [20,27,32]. The second system, SHP/Rgg_1299_, controls the transcription of the *shp_1299_* gene and a putative operon located downstream from the *rgg*_1299_ gene whose function remains undetermined [33]. Because little is known about the process by which the AIPs of the two SHP/Rgg systems and the ComS/ComR system are exported, we characterized the role of the PptAB exporter in these three QS systems with transcriptional fusions and a LC-MS/MS approach for three SHP_1358_, SHP_1299_ and SHP_279_. We used a transcriptomic approach to explore genome-level expression of the target genes in all functional RRNPP QS systems.

## 2. Materials and Methods

### 2.1. Bacterial Strains and Growth Conditions

Our study strain was the *S. thermophilus* wild-type strain LMD-9 (hereafter, the wild-type strain). It has two plasmids—plasmid 1 and plasmid 2 [34]. Table 1 lists the mutant strains that we constructed using this wild-type strain. *S. thermophilus* was grown at 37 °C or 42 °C in either M17 medium (Difco) supplemented with 10 g L^−1^ lactose (M17lac) or in a chemically defined medium (CDM) [35]. *Escherichia coli* was grown at 37 °C in Luria–Bertani broth with shaking. Agar (1.5%) was added to the media, as needed. When required, antibiotics were added to the media at the following final concentrations: erythromycin at 150 µg mL^−1^ for *E. coli* and at 5 µg mL^−1^ for *S. thermophilus* and kanamycin at 1 mg mL^−1^ for *S. thermophilus.*

### 2.2. DNA Manipulation and Sequencing

Restriction enzymes, T4 DNA ligase (New England Biolabs), and Phusion DNA polymerase (Finnzymes) were used in accordance with the manufacturers’ instructions. Standard methods were used to carry out DNA purification, restriction digestion, PCR, ligation, and sequencing. Table S1 lists the oligonucleotides we used (Eurofins). The *E. coli* strain TG1 *repA^+^* was used as the host for the cloning experiments [36]. *S. thermophilus* was transformed while using natural competent cells [36] with the addition of synthetic competence peptide (ComS, LPYFAGCL) at a concentration of 1 µM when required or using electrocompetent cells [20] when the strains where not naturally competent. The plasmids used are listed in Table S2.

### 2.3. Construction of the Mutant Strains

The overlapping PCR method was used to delete the *pptAB* genes and replace them with an erythromycin cassette (*erm*) as follows. The *erm* cassette was amplified via PCR with the oligonucleotides Erm-F and Erm-R and using pG^+^host9 as a template [37]; it was fused to fragments located upstream and downstream from the *pptAB* genes, also using PCR. The upstream and downstream fragments were amplified with the oligonucleotides pptABe_up-F/pptABe_up-R and pptABe_down-F/pptABe_down-R, respectively, while using chromosomal DNA from the wild-type strain as a template. The resulting PCR fragment was used to transform the wild-type strain, leading to the construction of TIL1486 strain (*pptAB::erm*). The TIL1488 (*blp::*P*_comX_-luxAB* *pptAB*::*erm*), TIL1489 (*blp*::P*_shp1358_-luxAB pptAB*::*erm*), and TIL1491 (*blp*::P*_shp1299_-luxAB aphA3 pptAB*::*erm*) strains were constructed by transforming the CB001, TIL1165, and TIL1038 strains, respectively, with chromosomal DNA from the TIL1486 strain.

We also constructed a mutant, the TIL1558 strain (Δ*pptAB*), in which we deleted an internal fragment of the *pptAB* genes via a double crossover event that involves the plasmid pG^+^host9. Thus, this mutant did not have an antibiotic resistance cassette. Briefly, the upstream and the downstream fragments of the *pptAB* genes were obtained via amplification using the primer pairs pptAB_up-F/pptAB_up-R and pptAB_down-F/pptAB_ down-R. The overlapping PCR method was used to fuse both fragments. The resulting fragment was double digested using the restriction enzymes EcoRI and KpnI and then ligated into pG^+^host9 between the EcoRI and KpnI restriction sites. The resulting plasmid, pG^+^host9::updown.*pptAB*, was used to transform competent cells of the wild-type strain. The integration and excision of this plasmid [37] then generated the TIL1558 strain. In this mutant, the promoter of the *pptA* gene (including its ATG) and the last 74 bp of the *pptB* gene were conserved.

The presence of the plasmid pBV5030::P_32_-*shp_1358_* allowed for the expression of the *shp_1358_* gene under the strong P_32_ promoter. This plasmid was used to transform the wild-type strain and the TIL1558 strain, resulting in the TIL1559 and TIL1563 strains, respectively.

A variety of strains were created in which the *comR* gene was replaced with a kanamycin resistance cassette. First, the upstream and downstream regions of the *comR* gene were amplified using the primers comR_up-F/comR _up-R and comR _down-F/comR _down-R, respectively. The kanamycin cassette (*aphA3*) was amplified via PCR using the primers AphA3-F/AphA3-R and plasmid pKa as the template [38]. This cassette was inserted between the upstream and downstream fragments of the *comR* gene via overlapping PCR. The resulting fragment was double digested with the restriction enzymes EcoRI and XhoI and then ligated into pG^+^host9 between the related restriction sites, creating the plasmid pG^+^host9::updown.*comRaphA3*. The linearized plasmid that was obtained via digestion with the restriction enzyme NdeI was used to naturally transform the wild-type, TIL773 (∆*eep*), and TIL1558 (∆*pptAB*) strains in order to generate the TIL1560 (*comR::aphA3*), TIL1561 (∆*eep comR::aphA3*), and TIL1562 (∆*pptAB comR::aphA3*) strains, respectively. The electrotransformation of the TIL883 strain (∆*amiCDE*) with the plasmid pG^+^host9::updown.*comRaphA3* and the subsequent processes of integration and excision generated the TIL1564 strain (∆*amiCDE comR::aphA3*).

In order to study the expression of the *pptAB* genes, a derivative of pGICB004a, pGICB004a::P*_pptAB_*, was constructed, as follows. The promoter of the *pptAB* genes was amplified using the oligonucleotides PpptAB-SpeI and PpptAB-EcoRI; double digested with the restriction enzymes SpeI and EcoRI; and finally, ligated into pGICB004a between the related restriction sites. ScaI-linearized pGICB004a::P*_pptAB_* was used to transform the wild-type strain, leading to the TIL1557 strain (*blp*::P*_pptAB_-luxAB aphA3*).

Finally, the plasmid pBV5030::P_32_ was used to transform the TIL1558 strain, resulting in the TIL1565 strain.

All of the constructions were verified by PCR and sequenced when necessary.

### 2.4. Mass Spectrometry Analysis

Liquid chromatography-tandem mass spectrometry (LC-MS/MS) was performed by coupling an LTQ-Orbitrap (Thermo Fisher Scientific, Waltham, MA, USA) with an UltiMate™ 3000 RSLCnano System (Thermo Fisher Scientific); analyses were carried out at the PAPPSO platform (MICALIS, INRAE, Jouy-en-Josas, France). The strains were grown in CDM until they reached an OD_600_ of 0.8, which corresponded to the end of the exponential growth phase. Three independent cultures (i.e., biological replicates) were grown for each strain studied. Supernatants were recovered via centrifugation (at 10,000 rpm and 4 °C for 10 min). Samples (4 µL) were loaded at 20 μL min^−1^ on a precolumn (µ-Precolumn, 300 µm × 5 mm, C18 PepMap100, 5 µm, 100 Å, Thermo Fisher) and then washed with loading buffer. After 3 min, the precolumn cartridge was connected to the separating column (Acclaim PepMap^®^, 75 μm × 500 mm, C18, 3 μm, 100 Å, Thermo Fisher). The flow rate was 300 nL min^−1^, and elution employed a linear gradient of acetonitrile (0.8% per min) in 0.1% formic acid. The eluted peptides were ionized with a spray voltage of 1.3 kV. A full MS scan (range: 400–2000 m/z) was performed while using the LTQ-Orbitrap. The full MS scans were automatically calibrated using the lock mass option and two dimethylcyclosiloxanes (391.284 and 536.165). The MS/MS analysis was carried out employing the collision-induced dissociation (CID) technique (40% collision energy), which was applied to three charge states (+1, +2, and +3) and the eight most abundant ions (dynamic exclusion of 30 s; minimal intensity threshold of 5 × 10^2^). We manually extracted the ion current signals (XIC) with Xcalibur Qual Browser (v. 3.0.63). We analyzed the masses of mature SHP_1358_ (EGIIVIVVG), 898.561 Da, SHP_1299_ (DIIIFPPFG), 1018.561 Da, SHP_279_ (EGIIVIGVG), 856.514 Da, and the streptide (AKGDGWKVM with a cyclization between the first K and the W), and 495.247 Da at a high level of resolution (10 ppm). The presence of these mature forms was confirmed when we detected at least three masses that corresponded to fragments that were produced by MS2 fragmentation (accuracy of 0.5 Da).

### 2.5. Luciferase Assays

**Figure 1:**
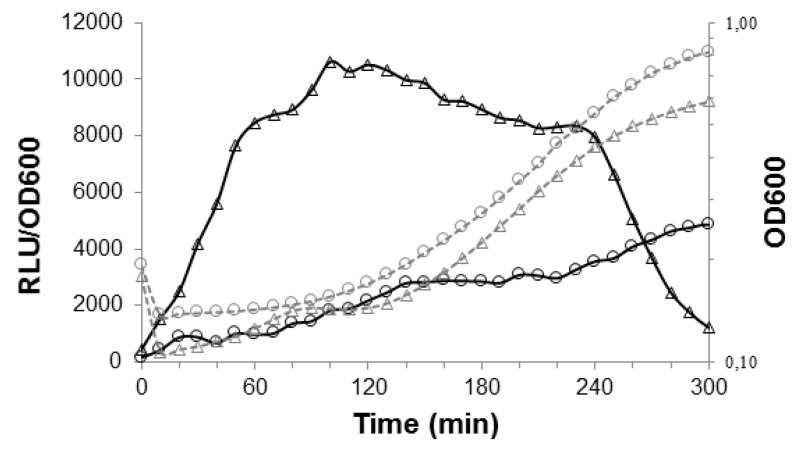
Growth and luciferase activity levels for the *S. thermophilus* TIL1557 strain (P*_pptAB_*-*luxAB*) grown in chemically defined medium (CDM) (Δ) versus M17lac (⚪). The growth curves (OD_600_) are depicted using gray dotted lines, and the relative levels of luciferase activity (RLU/OD_600_) are depicted using black solid lines. Data shown are representative of three independent experiments.

**Figure 2:**
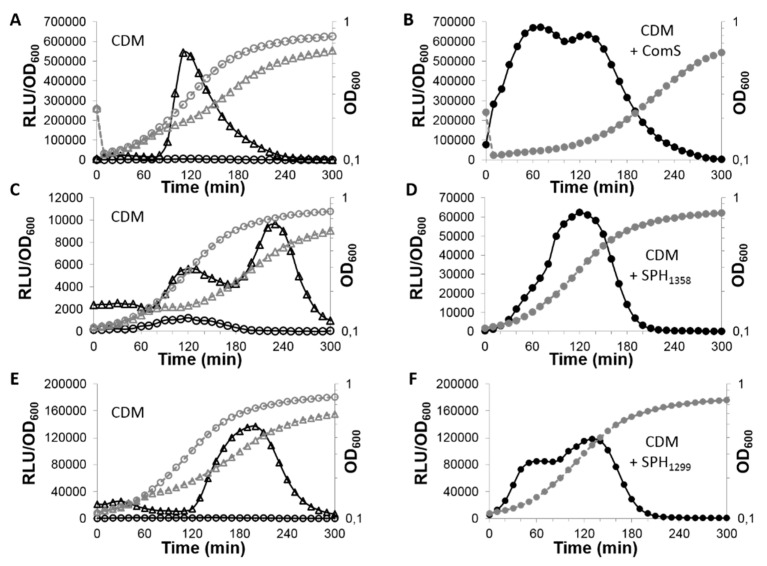
Growth and luciferase activity levels for strains that contained P*comX*-*luxAB*, P*shp_1358_*-*luxAB*, or P*shp_1299_*-*luxAB* fusions in a wild-type or a *pptAB*::*erm* background and grown in CDM with or without a synthetic autoinducing peptide (AIP). The growth curves (OD_600_) are presented in gray dotted lines, and the relative levels of luciferase activity (RLU/OD_600_) in black solid lines. Results are shown for strains grown in CDM containing P*comX*-*luxAB* (**A**), P*shp_1358_*-*luxAB* (**C**), or P*shp_1299_*-*luxAB* (**E**) fusions with a wild-type background (Δ) or *pptAB*::*erm* background (⚪) and for strains grown in CDM with the cognate AIP stated on the figure and containing P*comX*-*luxAB* (**B**), P*shp_1358_*-*luxAB* (**D**), or P*shp_1299_*-*luxAB* (**F**) fusions with a *pptAB*::*erm* background (●). Data shown are representative of three independent experiments.

For the *pptAB* genes expression study, TIL1557 was the strain used (Figure 1, Table 1). For the results that are described in Figure 2, strains used were CB001, TIL1488, TIL1165, TIL1489, TIL1038*,* and TIL1491 (Table 1). The luciferase assays were carried out, as follows. The cells were grown overnight at 37 °C in CDM or M17lac. The cultures were diluted to a final OD_600_ of 0.05 in 1 mL of CDM or M17lac. As necessary, synthetic peptides, SHP_1358_ (EGIIVIVVG), SHP_1299_ (DIIIFPPFG), or ComS (LPYFAGCL), prepared in DMSO for the two SHPs or water for ComS were added at a final concentration of 1 µM. We transferred 250 µL of these diluted cultures to the wells of a covered sterile white microplate with a transparent bottom (Greiner). The cultures’ OD_600_ and luminescence (as expressed in relative light units [RLU]) values were assessed at 37 °C in an Infinite M200 spectroluminometer (Tecan), as previously described [31]. 

### 2.6. Total RNA Extraction

The strains were grown in CDM at 42 °C, and samples were taken from the cultures at the end of the exponential growth phase (OD_600_ = 1). TIL1560, TIL1561, TIL1562, and TIL1564 were the strains used (Table 1). The cells were harvested via fast centrifugation (at 13,400 g for 15 s); the supernatants were removed; and, the bacterial pellets were frozen in liquid nitrogen and stored at −80 °C. The cells were then lysed in phenol-chloroform 5:1 (*v*/*v*) with a FastPrep FT120 tissue grinder (Thermo Savant, Holbrook, NY, USA). Two cycles of cell disruption were performed (both at 6.5 m s^−1^ for 45 s). Cellular debris was pelleted by centrifugation (at 16,000 g and 4 °C for 10 min.) and the supernatants were collected. The total RNA extraction was then performed using the Direct-Zol RNA MiniPrep Kit (Zymo Research, Irvine, CA, USA) in accordance with the manufacturer’s instructions. Genomic DNA was eliminated with a DNA-free DNA Removal Kit (Invitrogen, Waltham, MA, USA). Finally, RNA quantity and quality were assessed using a Qubit RNA HS Assay Kit (Thermo Fisher Scientific, USA) and a Bioanalyzer system (Agilent, Santa Clara, CA, USA), respectively. For each strain, RNA was extracted from the three biological replicates.

### 2.7. RNA Sequencing and Transcriptome Analysis

RNA sequencing was performed at I2BC (www.i2bc.paris-saclay.fr), a sequencing facility. The total transcript libraries were created using a Stranded mRNA Library Prep Kit (Illumina). We followed the manufacturer’s instructions, except in the initial steps (i.e., up until RNA fragmentation), where polyA purification was replaced by rRNA depletion using a Ribo-Zero Kit (Illumina). Quality control was carried out on the libraries using a Bioanalyzer HS DNA Kit (Agilent) and a dsDNA HS Assay Kit (Thermo Fisher Scientific, USA). The libraries were then sequenced (single-end reading, read length of 75 bp) using an Illumina NextSeq 500 System. The raw reads were demultiplexed, and fastq files were created from the output with bcl2fastq2 (v. 2.18.12; Illumina). The adapters were subsequently trimmed out of the reads using Cutadapt (v. 1.15; [39], and read quality was then verified via FastQC (v. 0.11.5; http://www.bioinformatics.babraham.ac.uk/projects/fastqc/). The clean reads in the total transcript libraries were then mapped against the *S. thermophilus* LMD-9 genome while using BWA-aln (v. 0.6.2-r126; [40]). The LMD-9 genome was processed beforehand using BactgeneSHOW to predict short CDSs [41,42]. We used the following option parameters: -m 4C_si -rbs m1 -duprev -cdst 0.01. The dataset included predicted CDSs (45–180 nt) that were not present in the GenBank annotation for LMD-9 (chromosome: CP000419.1; plasmid 1: CP000420.1; plasmid 2: CP000421.1) [34] (Supplementary Table S3). The raw read counts were calculated using the featureCounts tool (option –s 1) in the SubRead package (v. 1.5.2; [43]). We employed DESeq2 (v. 1.18.1; [44]) to carry out normalization and differential analysis. First, the total read counts were normalized using the median of ratios method [45]. Subsequently, gene expression was compared between our reference strain, TIL1560 (*comR::aphA3*), and the TIL1561 (Δ*eep comR::aphA3*), TIL1562 (Δ*pptAB comR::aphA3*), and TIL1564 (Δ*amiCDE comR::aphA3*) strains to examine the roles of the *eep*, *pptAB*, and *amiCDE* genes. The resulting *p*-values were adjusted using the false discovery rate method [46]. In our further analyses, we only focused on genes with adjusted *p*-values ≤ 0.01 and for which there was at least a 1.9-fold difference between the reference strain and the other strains. The RNA-seq raw data from this study have been deposited in the ArrayExpress database at EMBL-EBI under accession number E-MTAB-9568 (www.ebi.ac.uk/arrayexpress).

### 2.8. Bioinformatic Tools

We employed BlastP (v. 2. 9.0) with standard parameters and the PptAB proteins of *S. mutans* UA159 as baits to identify the PptAB homologous proteins of *S. thermophilus* LMD-9. PptA of *S. thermophilus* was found to be encoded by the Ster_RS07730 gene (previous nomenclature: Ster_1572). PptB of *S. thermophilus* was found to be encoded by the Ster_RS07725 gene (previous nomenclature: Ster_1571). We used the 65 complete genome sequences available in the NCBI database (https://www.ncbi.nlm.nih.gov/genome) to compare the amino acid sequences of PptA and PptB inside the *S. thermophilus* species.

## 3. Results

### 3.1. The PptAB Genes Are Expressed in S. thermophilus and Required for the Positive Control of Three Signaling Systems

PptAB is an ABC transporter that is made of a membrane-bound cytoplasmic ATP-binding protein, PptA, which provides the energy for substrate translocation, and a transmembrane protein, PptB, which forms the pore. It was first identified in *E. faecalis* [19]. However, shortly thereafter, orthologous genes encoding exporters with similar functions were found in *S. pyogenes*, *S. mutans* [30] and *S. agalactiae* [24]. We used the PptAB proteins in the *S. mutans* UA159 strain to identify any corresponding proteins in the *S. thermophilus* LMD-9 strain [47]. PptA of *S. thermophilus* shared 77% identity with PptA of *S. mutans* (88% sequence similarity) and PptB shared 51% identity with PptB of *S. mutans* (70% sequence similarity). The PptA and PptB proteins are highly conserved in the species *S. thermophilus*, estimates of shared identity range between 98 and 100 for PptA and between 97 and 100 for PptB.

First, we characterized the expression patterns of the *pptAB* genes in two media, CDM and M17lac, while using a P*_pptAB_*-*luxAB* transcriptional fusion in the TIL1557 strain. We found that gene expression was constitutive throughout the exponential growth phase and it was higher in CDM than in M17lac (Figure 1). Second, we deleted the *pptAB* genes and characterized the phenotype of the resulting strains. To date, three RRNPP signaling systems have been studied in the LMD-9 strain of *S. thermophilus*. They involve three different AIPs that are potentially exported by PptAB, but only two systems have been associated with a phenotype.

The first is a SHP/Rgg system that is based on a nine-amino-acid-long hydrophobic AIP, SHP_1358_ (EGIIVIVVG). This system helps to produce the streptide encoded by the Ster_RS010575 gene [27]. Therefore, we measured streptide production by the TIL1179 strain, which had a wild-type background and the TIL1565 strain, which bore the Δ*pptAB* mutation using LC-MS/MS; streptide levels were quantified by measuring the integrated area under the curve in extracted-ion chromatograms for the three biological replicates per strain. The TIL1179 strain had a mean (±SD) streptide level of 2.7 × 10^8^ (±4 × 10^7^). In contrast, the streptide level was < 7 × 10^4^ in the TIL1565 strain. This result indicates that streptide was only produced at trace levels when the *pptAB* genes were inactivated.

The second system is based on the mature form of ComS (IAILPYFAGCL), which is also hydrophobic, but composed of eleven amino acids [28]. It controls the activity of ComR, a transcriptional regulator that is involved in mediating competence. Indeed, the ComS/ComR system positively controls the transcription of the *comX* gene. ComX is an alternative sigma factor that controls the transcription of genes encoding the transformasome, a protein complex essential for competence [31,48]. Therefore, we assessed the competence of the TIL1558 strain, a Δ*pptAB* mutant, when it was grown in CDM with pG^+^host9. No transformants were detected, which indicated that the mutant had completely lost the ability to transform naturally. However, the transformability of strain TIL1558 was restored with the addition of synthetic ComS in the CDM medium, as demonstrated with the construction of strains TIL1562, TIL1563, and TIL1565 (Table 1).

The third RRNPP system is the SHP/Rgg_1299_ system. It is known to be functional, but its target function is unknown.

Thus, we wanted to demonstrate that streptide production and competence were specifically influenced by the elimination of expression of the RRNPP QS target genes in the Δ*pptAB* mutant. Therefore, we quantified the expression of the *comX* gene, a target of the ComS/ComR system, the *shp_1358_* gene, a target of the SHP/Rgg_1358_ system, and also the *shp_1299_* gene, a target of the SHP/Rgg_1299_ system. The mature form of its AIP, SHP_1299_ (DIIIFPPFG), is similar to SHP_1358_ because it is also nine amino acids long and mostly hydrophobic. To this end, we used transcriptional fusions between the promoter of these three genes and the *luxAB* genes encoding a luciferase and then explored gene expression in the wild-type versus the *pptAB::erm* background. In the three systems, the genes encoding the AIPs, *shp* genes, or *comS* gene, are system targets, creating a positive feedback loop and resulting in the marked expression of all the target genes [27,31,33]. Interestingly, although the three systems function similarly because they involve the export and reimport of AIPs, the three target genes were not expressed at the same moment during strain growth (Figure 2). The *comX* gene and the *shp*_1299_ gene were expressed at the beginning and the middle of the exponential growth phase, respectively (Figure 2A,E). In contrast, the *shp*_1358_ gene displayed a bimodal pattern during growth: there was a lower peak of expression early on and then a higher peak at the end (Figure 2C). These differences in the timing of expression indicate that, although the mechanisms that underlie each system are similar, they are not identical. In the *pptAB::erm* background, the expression of the *comX* and the *shp*_1299_ genes was completely absent (Figure 2A,E) and it dropped to trace levels for the *shp*_1358_ gene. In conclusion, these results show that PptAB is necessary for the expression of the three target genes and, therefore, for the functionality of the three QS systems.

We added the cognate mature AIPs ComS, SHP_1358_, and SHP_1299_ to cultures of the strains that contained the three transcriptional fusions in the *pptAB*::*erm* background in order to verify PptAB’s predicted role in AIP export. The addition of the cognate AIPs immediately triggered the expression of the *comX*, or *shp*_1299_ genes at levels that are similar than those obtained when the *pptAB* genes were functional (Figure 2B,F) and at a level six times higher for the *shp*_1358_ gene (Figure 2D). Consequently, the functionality of the three QS systems was fully restored, confirming that PptAB plays a role in signal production, not signal detection, for these three systems. We deleted the *amiCDE* genes in strains TIL1488 (*blp*::*comX*-*luxAB pptAB*::*erm)*, TIL1489 (*blp*::*Pshp_1358_*-*luxAB pptAB*::*erm)*, and TIL1491 (*blp*::*shp_1299_*-*luxAB pptAB*::*erm*). Using luciferase assays, we checked that the expression of the *comX*, *shp_1358_* and *shp_1299_* genes remained absent in the presence of the cognate AIP (data not shown), which confirmed that Ami is the importer of the AIPs in strain TIL1488, TIL1489, and TIL1491.

### 3.2. PptAB is Involved in the Export of SHP_1358_ and SHP_1299_

To determine whether PptAB helps export the AIPs in *S. thermophilus*, we used LC-MS/MS to ascertain whether mature forms of SHP_1358_ and SHP_1299_ were present in the wild-type strain and absent in the Δ*pptAB* mutant (TIL1558). We did not look for ComS, which is not detectable via LC-MS/MS in the supernatant of the wild-type strain [28].

As expected based on prior research, we found mature forms of SHP_1358_ and SHP_1299_ in the wild-type strain [27,33] (Figure S1) but not in the Δ*pptAB* mutant. This result fits with our prior research showing that the *shp*_1358_ and *shp*_1299_ genes were not expressed in the *pptAB* mutant due to the disruption of the positive feedback loop.

To further characterize the key role played by PptAB as a peptide exporter, we delved deeper into how the mature form of SHP_1358_ is produced. We uncoupled the expression of the *shp*_1358_ gene from the presence of PptAB by introducing the plasmid pBV5030::P32-*shp*_1358_ into the Δ*pptAB* mutant, creating the TIL1563 strain. Because of the plasmid, *shp*_1358_ expression was under the control of a strong promoter and it was not reliant on the autoregulation loop generated by the SHP/Rgg_1358_ system. As a result, there was constant independent synthesis of a SHP_1358_-like precursor, which, due to technical constraints, contained a glycine residue between the methionine at position 1 and the lysine at position 2 of the wild-type sequence [27]. This 24-amino-acid-long precursor (hereafter, SHP*_1358_) can serve as the basis for the 9-amino-acid-long mature wild-type peptide, as seen below in the results for the TIL1213 strain. We did not detect the presence of mature SHP_1358_ in the TIL1563 strain, which demonstrated that the PptAB transporter is required for the peptide’s production.

### 3.3. PptAB Only Exports the Mature Form of SHP_1358_

Past research has shown that PptAB is necessary for the production of active AIPs, but which form(s) of AIPs are recognized by PptAB? We cannot exclude the possibility that PptAB can export forms that are longer than mature AIPs, which may undergo further maturation via the action of extracellular proteases. PptAB might also be able to export the complementary sequence of the mature form of SHP*_1358_ (i.e., the N-terminal peptide that results from the maturation of SHP*_1358_ [SHP*_1358 (1–15)_]) and the related products of degradation. Therefore, using the TIL1213 strain (Δ*amiCDE* pBV5030::P_32_-*shp_1358_*), we looked for traces of SHP*_1358 (1–15)_ as well as for fragments that result from shortening at the N-terminus (from SHP*_1358 (1–15)_ to SHP*_1358 (7–15)_) and at the C-terminus (from SHP*_1358 (1–14)_ to SHP*_1358 (1–10)_) (Table S4). The TIL1213 strain might have accumulated these peptides, since it was able to synthesize the AIP precursor thanks to the presence of the plasmid pBV5030::P_32_-*shp*_1358_; however, the strain was unable to reimport any peptides because the Ami importer was inactivated. While we confirmed the presence of mature SHP_1358_ in the supernatant, we found no evidence of the fragments (data not shown). These results indicate that the precursor is likely matured in either the cytoplasm or in the cell membrane prior to secretion and that PptAB only interacts with the peptide’s mature form.

### 3.4. PptAB Controls Transcription of the Genes Located Downstream from the Rgg Genes in S. thermophilus

**Figure 3:**
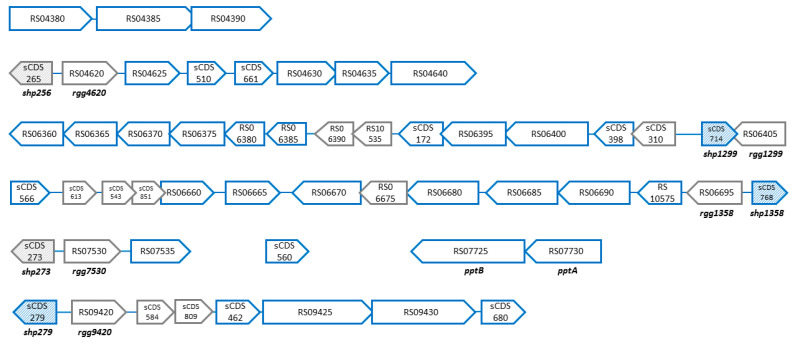
Organizational schematic of the genes controlled by PptAB. The genes whose expression was upregulated in the TIL1560 strain (*comR*::*aphA3*) compared to the TIL1562 strain (*comR*::*aphA3* Δ*pptAB*) are shown in blue. This set of genes was controlled by PptAB but did not include genes regulated by ComR, which controls competence. ORF orientation within each cluster is indicated by the direction of the arrowhead (not to scale). Genes encoding SHPs have hatched shading. Genes that were not regulated by PptAB are in gray. Genes were named and described in accordance with their GenBank annotations, to which we added in-lab annotations for the short genes (see the Materials and methods section and Table S3).

Because PptAB helps export the AIPs that control the activity of transcriptional regulators, we used a deep sequencing approach (RNAseq) to identify all of the genes whose expression is controlled by PptAB. The genome of LMD-9 contains three complete *shp*/*rgg* loci in addition to the *shp*/*rgg_1358_* and *shp*/*rgg_1299_* loci, as well as a locus with truncated *shp* and *rgg* genes (sCDS_305/Ster_RS03240) (Figure S2A). Thus, we hypothesized that other genes in the near or far vicinity of the *rgg* genes could also be controlled by this transporter. The ComR regulon, partially controlled by PptAB through the export of the mature form of its cognate AIP ComS, was previously identified in *S. thermophilus* [26,48]. Because the regulon encompasses more than 100 genes, we chose to work in a Δ*comR* background, so that we could identify PptAB regulon in the absence of the genes that are regulated by the ComS/ComR system. Therefore, we compared gene expression between the TIL1560 (*comR::aphA3*) and TIL1562 (*comR*::*aphA3* Δ*pptAB*) strains. The *shp*_1299_ and *shp*_1358_ genes, the two distinct targets of the SHP/Rgg systems, were highly expressed at the end of the exponential growth phase (Figure 1), so samples for mRNA extraction were obtained at this stage (OD_600_ = 1). Genes were upregulated in the TIL1520 strain compared to in the TIL1562 strain (Table 2; Figure 3). Except sCDS_305/Ster_RS03240 locus (Figure S2A), the expression of all genes located downstream of the other five *rgg* genes and some, but not all of their cognate *shp* genes were controlled by PptAB. Hereafter, these genes will be referred as the proximal target genes of the Rgg regulators. We discovered two distal target genes (i.e., not located near a *shp*/*rgg* locus) that were regulated by PptAB: one short CDS (CDS_560) that was poorly expressed and one operon (Ster_RS04380-90). The short CDS has an unknown function, and the operon contains genes that are annotated as encoding enzymes that are involved in cysteine metabolism.

We could distinguish three classes of genes located downstream from the *rgg* genes. First, there were two sets of highly expressed genes (based on the average of the normalized read counts) located downstream from the two *rgg* genes already studied, *rgg*_1299_ and *rgg*_1358_; they were strongly controlled by PptAB and displayed greater than 45-fold changes in expression (Table 2). The genes downstream from the *rgg*_1299_ gene were all transcribed in the same direction, but, the greater their proximity, the higher their level of transcription and fold change. The operon’s initial genes encode an enzyme (annotated oligopeptidase F), a transporter, and a peptide. The function of these genes is unknown. The operon’s later genes encode two peptides, an ABC transporter, and a two-component system whose function seems to be linked to rhamnose-glucose polysaccharide synthesis induced by bacitracin stress [49]. The genes downstream from *rgg*_1358_ form two transcriptional units. The first unit was transcribed in the same direction as *rgg*_1358_ and it was strongly controlled by PptAB; it encodes proteins that are involved in the production of the streptide [20,32] and two additional proteins with unknown functions. The second unit was transcribed in the opposite direction and it was weakly controlled by PptAB; it encodes peptides or small proteins with unknown functions. The transcription of the two *shp* genes at these loci was also strongly controlled by PptAB, as had already been demonstrated via the transcriptional fusions with the luciferase reporter (Figure 2).

The second class of genes involved two *shp*/*rgg* loci, sCDS_273/Ster_RS07530 and sCDS_279/Ster_RS09420. For this gene class, gene expression was relatively high in the TIL1560 strain, with fold changes ranging between 13 and 29. The *shp* gene at the sCDS_273/Ster_RS07530 locus was not expressed. The *shp* gene at the sCDS_279/Ster_RS09420 locus was weakly expressed and weakly controlled by PptAB (1.9-fold change). The sole proximal target gene in the sCDS_273/Ster_RS07530 system encodes a transporter with an unknown function. The proximal target genes in the sCDS_279/Ster_RS09420 system are similar to those at the streptide locus, with a gene encoding a SAM radical enzyme, a gene encoding a transporter, and two genes encoding peptides with unknown functions. The third class of genes was associated with the locus sCDS_265/Ster_RS04620. The genes were weakly expressed and controlled by PptAB (three-fold change). The *shp* gene at this locus was not expressed. Again, the proximal target genes were similar to those at the streptide locus, with a gene encoding a SAM radical enzyme, a gene encoding a transporter, two genes encoding peptides, a gene encoding an Fe-S oxido-reductase, and a gene encoding an arginase.

We found very similar expression levels and fold changes when we compared gene expression across the TIL1560 (*comR::aphA3*), TIL1561 (Δ*eep comR::aphA3*), and TIL1564 (Δ*amiCDE comR::aphA3*) strains (Tables S5 and S6). The differences mainly involved short CDSs encoding genes that were poorly expressed and for which regulation resulted in a three-fold change at most (sCDS_180, sCDS_560, sCDS_279, and sCDS_854). Only one gene (Ster_RS06940) was found to be downregulated in the *eep* mutant (Table S5); it encodes a peptide-binding protein of the Ami transporter and it was expressed at high levels in the TIL1560 strain. The expression of this gene was unaffected by the *amiCDE* and *pptAB* mutations. We also found that one gene (Ster_RS09420) was upregulated 1.9 fold in the *amiCDE* mutant, but it was poorly expressed in general (data not shown). Furthermore, it is important to note that none of the genes in plasmid 1 were expressed in the TIL1561 strain. Upon PCR verification, we discovered that this strain had lost this plasmid during the construction process (data not shown).

Finally, we observed that the *shp_279_* gene was expressed at very low levels. As a result, we questioned whether the expression levels were sufficient to allow for the production of mature SHP_279_ in the wild-type strain. We found that the mature form of SHP_279_ was indeed present in the wild-type strain (Figure S3) as well as in the TIL883 strain (Δ*amiCDE*) (data not shown). Interestingly, it was absent in the TIL773 (Δ*eep*) and TIL1558 (Δ*pptAB*) strains.

## 4. Discussion

PptAB has been identified as the exporter of the pheromones of PrgX in *E. faecalis* [19] and of Rgg and ComR in pathogenic streptococci *S. pyogenes*, *S. mutans* [30] and *S. agalactiae*, [24]. PptAB also exports peptides in *S. aureus* via a mechanism that is similar to the one that produces the pheromones in *E. faecalis.* However, the biological function of these peptides remains unclear [50,51]. The role of PptAB in the starter species *S. thermophilus* had previously remained unexplored, even though this bacterium has a *comS*/*comR* locus and it is one of the streptococcus species with *S. pneumoniae* with the greatest number of paralogous *shp*/*rgg* loci. In this study, we used genetic approaches and mass spectrometry to show that PptAB can export the mature AIPs of four different systems: ComS/ComR, SHP/Rgg_1299_, SHP/Rgg_1358_, and SHP/Rgg_9420_. Focusing on the SHP/Rgg_1358_ system, we found direct evidence, namely the presence of mature SHP_1358_, that PptAB acts as a peptide exporter of this specific peptide.

Here, we studied the mature forms of AIPs in *S. thermophilus*. These peptides are hydrophobic and short in length, ranging in size from mime amino acids (the SHPs) to 11 amino acids (the mature ComS). These characteristics are shared by PptAB-transported AIPs in other streptococci. In the wild-type strain, we found no traces of the complementary part of mature SHP_1358_, a short hydrophobic peptide (14 amino acids long) that should be released after the cleavage of the precursor; we also did not observe any fragments of this complementary part. This result raised questions regarding the specificity of the transporter. One hypothesis is that PptAB only exports mature SHP_1358_ because its activity focuses on mature AIPs. Another hypothesis is that there is interplay between PptAB and the protease Eep, which is known to be involved in the maturation of these peptides [21,24,27]. Eep is a transmembrane zinc-metalloprotease whose active cleavage site is thought to occur in a transmembrane domain [52]. It is possible that only mature SHPs are directly delivered to PptAB. If such is the case, the fate of the complementary part remains unknown.

Most commonly, AIPs of Gram-positive bacteria are exported either by the general Sec-dependent pathway or by dedicated peptidase-containing ABC transporters that can export and mature double glycine signaling peptides, which play a role in competence or provide competitive fitness advantages in complex environments [26,53,54]. The PptAB export mechanism represents an intermediate solution that is neither highly generalized nor highly specific. Further research should explore the potential evolutionary benefits of this solution and, more importantly, assess whether it is a point of control in QS systems. While the expression of the *pptAB* genes seems constitutive, more work is needed to determine whether PptAB activity is somehow regulated.

The RNAseq approach was used to identify the genes whose expression was controlled by PptAB, Eep, and AmiCDEF. All of the genes located downstream from the five non truncated *rgg* gene are part of the targets genes. Our findings suggest that non-truncated *shp*/*rgg* loci are functional and involved in the actions taken by PptAB, Eep, and AmiCDEF to regulate these target genes. Nevertheless, further work is needed in order to confirm that the expression of these target genes is indeed controlled by their cognate *rgg* gene. These target genes were generally expressed and regulated at similar levels by the three proteins PptAB, Eep, and AmiCDEF, indicating that these target genes might belong to a single transcriptional unit. There were a few exceptions: for example, when the proximal targets of SHP/Rgg_1299_ were examined, the genes farthest from the *rgg* gene (Ster_RS06385- Ster_RS06360) were poorly expressed and regulated. However, this effect may have resulted from readthrough transcription dynamics, rather than from issues with QS regulation. Surprisingly, these genes are also part of the ComR regulon [26]. Although some of these loci had been previously studied, using the RNAseq approach, we were able to identify additional proximal targets, such as Ster_RS06680 and Ster_RS06670 for the *shp*/*rgg*_1358_ locus, and Ster_sCDS172 for the *shp*/*rgg*_1299_ locus. Ster_RS06680 and Ster_RS06670 have been annotated as SPASM-domain-containing protein and sodium transporters, respectively. Here, we discovered that they belong to the *str* operon, but further work is needed in order to clarify their role in streptide production. The function of Ster_sCDS172 is also unknown. We additionally found significantly expressed distal target genes with a three-gene operon linked to cysteine synthesis. Research is currently underway to identify the SHP/Rgg system that is associated with this operon and to confirm the operon’s function. To our knowledge, it is one of the few distal target gene of a SHP/Rgg system identified to date, although we must still verify that transcriptional control is direct. Indeed, few RNAseq approaches have been developed to exhaustively identify SHP/Rgg system targets in streptococci. These experiments highlighted genes located near the *rgg* genes in *Streptococcus mitis* [55], *Streptococcus pneumoniae* [23], or *S. pyogenes* [21].

The identity and expression patterns of those genes regulated by PptAB, Eep, and Ami were strikingly similar. This finding indicates that an equivalent role is being played in export, maturation, and reimport. It is highly likely that the deletion of these genes would completely inactivate the mechanism in action, leading to similar results for target gene transcription. However, a closer look at the expression of the *shp* genes in the wild-type strain versus the Δ*pptAB*, Δ*eep*, and Δ*amiCDE* background revealed the existence of three different regulation scenarios.

First, the *shp*_1299_ and *shp*_1358_ genes were expressed at high levels, and their expression was clearly controlled by the three proteins. The result was a positive feedback loop and marked expression of the proximal target genes at the end of the exponential growth phase in the wild-type strain background. These results correspond to a scenario, in which SHP is exported by PptAB, matured by Eep, and reimported by Ami. Consequently, the activity of the associated Rgg regulator is controlled, and there is the transcription of the genes located downstream from the *rgg* gene.

Second, the *shp*_273_ and *shp*_265_ genes were not expressed. This result suggests that a SHP encoded by another locus controls the activity of Rgg_7530_ and Rgg_4620_. We feel that there is more support for this hypothesis than for a hypothesis that involves the involvement of a non-cognate SHP/Rgg complex, because all of the *rgg* genes in the wild-type strain were expressed at similar levels indicating that the genes are likely functional also this deserves further validation. The proximal target gene in the SHP_273_/Rgg_7530_ system was expressed at high levels and regulated by PptAB, Eep, and Ami, whereas the proximal target genes of SHP_265_/Rgg_4620_ were expressed at lower levels and subject to less regulation by the three proteins. Interestingly, SHP_273_ is nearly identical to SHP_1358_: there is a single amino acid difference, a spot occupied by a valine in SHP_1358_ is occupied by a leucine in SHP_273_. SHP_265_ and SHP_1358_ differ by two amino acids: spots occupied by a glycine and a leucine in SHP_1358_ are occupied by a serine and an alanine, respectively, in SHP_265_ (Figure S2B). This finding suggests that SHP_1358_ is a good candidate for interacting with Rgg_7530_ and Rgg_4620_, and it could more effectively cross-activate Rgg_7530_, but less effectively cross-activate Rgg_4620_, if we assume that the cognate SHP of a Rgg is likely to be the most efficient SHP. Further experimental work is needed in order to confirm that SHP_1358_ can interact with different Rggs.

Third, the gene encoding SHP_279_ was poorly expressed and controlled by PptAB, Eep, and Ami, but the mature form of SHP_279_ was present in the wild-type strain background. In contrast, the system’s proximal target genes were expressed at higher levels and more strongly regulated by these three proteins than the *shp*_279_ gene. The Rgg regulator that was encoded by Ster_RS09420 plays a role in oxidative stress defense in the *S. thermophilus* CNRZ368 strain [56]. This system did not display a positive feedback loop related to *shp*_279_ expression, providing an additional element of support for the hypothesis that another SHP might interact with the Rgg_9420_ regulator. SHP_279_ is nearly identical to SHP_1358_: a spot that is occupied by a valine in SHP_1358_ is occupied by a glycine in SHP_279_. Consequently, SHP1358 is again a good candidate for Rgg_9420_ activation.

In a previous study, we demonstrated the absence of cross-activation between the SHP_1299_/Rgg_1299_ and SHP_1358_/Rgg_1358_ systems [33]. This result was expected, since the amino acid sequences of the two SHPs are not similar. Our findings here suggest that some SHP/Rgg systems can cross-activate each other, likely via highly similar SHPs, although this hypothesis must be experimentally tested. However, the fact that some SHP/Rgg loci encoded *shp* genes that were not expressed and that appeared to be activated by SHPs from other systems was surprising. Therefore, we wonder if, in *S. thermophilus*, these accumulated SHP/Rgg systems have degenerated or if they may prove to be useful under certain natural conditions. We also cannot exclude the possibility that other growth conditions exist, in which these *shp* genes are efficiently expressed. Previous work has shown that intracellular cross-activation involving 2 SHP/Rgg systems exists in *S. pneumoniae* [25] and in *S. pyogenes* [21]. However, circumstances are slightly different in the latter case because the two SHPs can interact with two Rggs with relatively similar affinities [57], although one Rgg activates transcription whereas the other inhibits it [21]. Another level of complexity is present if we consider interspecies crosstalk. This cross-talk can occur between streptococci species via SHP/Rgg systems, as demonstrated between *S. pneumoniae* and *S. mitis* [55], but also between distinct species via another RRNPP mechanism between *S. aureus* and *E. faecalis*. *S. aureus* can produce at least four linear peptides during the maturation process of secretion signal peptides of lipoproteins; one of these peptides can mimic a pheromone of *E. faecalis* and cause aggregation, the first step in horizontal gene transfer in this species [50]. Interestingly, in *S. aureus*, the transporter EcsAB, encoded by paralogs of the genes that encode PptAB and Eep, are necessary for the production of these peptides [50,51].

## 5. Conclusions

In conclusion, in *S. thermophilus*, PptAB plays a central role in the export of the AIPs that are involved in intracellular QS mechanisms. A RNAseq approach designed to identify the genes regulated by the exporter PptAB, the protease Eep and the importer Ami highlighted cross-talk between SHP/Rgg systems. More work is needed now in order to dissect this crosstalk at the strain level, but also at intra- an inter-species levels and to understand their various roles in natural and complex environments.

## Figures and Tables

**Table 1:** *S. thermophilus* strains used in this study.

Strain	Genotype	Resistance ^a^	Description ^b^	Source or Reference
LMD-9	*wild-type*			[34]
CB001	*blp::*P*_comX_-luxAB*			[31]
TIL773	Δ*eep*			[27]
TIL883	Δ*amiCDE*			[20]
TIL1038	*blp*::P*_shp1299_-luxAB aphA3*	Km		[33]
TIL1165	*blp*::P*_shp1358_-luxAB*			[27]
TIL1179	pBV5030::P_32_	Er		[28]
TIL1213	Δ*amiCDE* pBV5030::P_32_-*shp_1358_*	Er		[27]
TIL1486	*pptAB*::*erm*	Er	PCR fragment *pptAB::erm* → LMD-9	This study
TIL1488	*blp::*P*_comX_-luxABpptAB*::*erm*	Er	TIL1486 DNA → CB001	This study
TIL1489	*blp*::P*_shp1358_-luxAB pptAB*::*erm*	Er	TIL1486 DNA → TIL1165	This study
TIL1491	*blp*::P*_shp1299_-luxAB aphA3 pptAB*::*erm*	Er Km	TIL1486 DNA → TIL1038	This study
TIL1557	*blp*::P*_pptAB_-luxAB aphA3*	Km	pGICB004a::P*_pptAB_* → LMD-9	This study
TIL1558	Δ*pptAB*		pG^+^host9::updown.*pptAB* → LMD-9	This study
TIL1559	pBV5030::P_32_*-shp_1358_*	Er	pBV5030::P_32_-*shp_1358_* → LMD-9	This study
TIL1560	*comR::aphA3*	Km	pG^+^host9::updown.*comRaphA3* → LMD-9	This study
TIL1561	Δ*eep comR::aphA3*	Km	pG^+^host9::updown.*comRaphA3* → TIL773	This study
TIL1562	Δ*pptAB comR::aphA3*	Km	pG^+^host9::updown.*comRaphA3* → TIL1558	This study
TIL1563	Δ*pptAB* pBV5030::P*_32_*-*shp_1358_*	Er	pBV5030::P_32_-*shp_1358_* → TIL1558	This study
TIL1564	Δ*amiCDE comR::aphA3*	Km	pG^+^host9::updown.*comRaphA3*. → TIL883	This study
TIL1565	Δ*pptAB* pBV5030::P_32_	Er	pBV5030::P_32_ → TIL1558	This study

^a^ Km and Er = resistance to kanamycin and erythromycin, respectively. ^b^ The arrows indicate construction via transformation with chromosomal DNA or a plasmid.

**Table 2:** Genes significantly upregulated in the TIL1560 strain (comR::aphA3) versus in the TIL1562 strain (comR::aphA3 ΔpptAB) (positively controlled by PptAB).

Locus Tag	Old Locus Tag	Fold Change	TIL1560 *	TIL1562 *	Start	Stop	Strand	Gene Name	Protein Length	Description
**Cluster STER_RS04380-STER_RS04390**										
STER_RS04380	STER_0885	3.6	2552	704	817570	818481	+		303	cysteine synthase family protein
STER_RS04385	STER_0886	3.8	3818	1006	818503	819687	+		394	aminotransferase class V-fold PLP-dependent enzyme
STER_RS04390	STER_0887	3.9	1704	433	819629	820210	+		193	serine acetyltransferase
**Locus sCDS_265/Ster_RS04620**										
STER_sCDS_265	STER_0933	NR	20	7	864863	864934	−	*shp_265_*	23	
STER_RS04620	STER_0934	NR	1214	1296	865021	865878	+	*rgg_4620_*	285	MutR family transcriptional regulator
STER_RS04625	STER_0935	3.0	3966	1322	865938	866738	+		266	Fe-S oxidoreductase
STER_sCDS_510		3.8	990	259	866798	866947	+		49	
STER_sCDS_661		2.5	351	141	867030	867119	+		29	
STER_RS04630	STER_0937	2.7	3087	1132	867149	868246	+		365	radical SAM protein
STER_RS04635	STER_0938	2.8	1748	629	868243	868986	+		247	arginase family protein
STER_RS04640	STER_0939	2.2	3534	1641	868993	870165	+		390	MFS transporter
**Locus shp/rgg_1299_**										
STER_RS06360	STER_1290	2.0	601	296	1198824	1199426	−	*rr06*	200	response regulator transcription factor
STER_RS06365	STER_1291	1.9	1125	593	1199423	1200517	−	*hk06*	364	sensor histidine kinase
STER_RS06370	STER_1292	11.2	504	45	1200510	1201247	−		245	ABC transporter permease
STER_RS06375	STER_1293	12.8	545	43	1201244	1202125	−		293	MULTISPECIES: ABC transporter ATP-binding protein
STER_RS06380	STER_1294	13.3	111	8	1202118	1202297	−		59	hypothetical protein
STER_RS06385	STER_1295	18.7	183	10	1202308	1202517	−		69	hypothetical protein
STER_RS06390		ND	ND	ND	1202668	1202934	−		87	hypothetical protein (incomplete)
STER_RS10535		ND	ND	ND	1202925	1203152	−		74	hypothetical protein (incomplete)
STER_sCDS_172		56.6	8877	157	1203195	1203290	−		31	
STER_RS06395	STER_1296	59.7	93273	1563	1203277	1204506	−		409	MFS transporter permease
STER_RS06400	STER_1297	49.7	195871	3942	1204484	1206817	−		777	oligoendopeptidase F
STER_sCDS_398		46.3	97	2	1206867	1206920	−		17	
STER_sCDS_310		NR	1	1	1206895	1206960	−		21	
STER_sCDS_714	STER_1298	67.4	1015	15	1207133	1207213	+	*shp1299*	26	
STER_RS06405	STER_1299	NR	924	1060	1207191	1208066	−	*rgg1299*	291	XRE family transcriptional regulator
**Locus shp/rgg_1358_**										
STER_sCDS_566		9.8	76	8	1255722	1255835	+		37	
STER_sCDS_613		NR	25	31	1256064	1256165	+		33	
STER_sCDS_543		NR	26	14	1256228	1256353	+		41	
STER_sCDS_851		NR	0	0	1256331	1256390	+		19	
STER_RS06660	STER_1350	3.1	137	44	1256335	1256604	+		89	hypothetical protein
STER_RS06665	STER_1351	NR	55	70	1256764	1257063	+		99	hypothetical protein
STER_RS06670	STER_1352	114.6	32175	281	1257163	1258074	−		303	sodium transporter
STER_RS06675		ND	ND	ND	1258071	1258393	−		107	hypothetical protein (incomplete)
STER_RS06680		132.9	21819	164	1258390	1258992	−		200	SPASM domain-containing protein
STER_RS06685	STER_1355	113.3	31202	275	1259086	1260177	−	*strC*	363	MULTISPECIES: transporter
STER_RS06690	STER_1356	109.6	53740	490	1260174	1261493	−	*strB*	439	KxxxW cyclic peptide radical SAM maturase
STER_RS10575	STER_1357	78.0	6544	84	1261563	1261655	−	*strA*	30	KxxxW-cyclized peptide pheromone
STER_RS06695	STER_1358	NR	450	565	1261738	1262598	−	*rgg1358*	286	MutR family transcriptional regulator
STER_sCDS_768		114.8	723	6	1262689	1262760	+	*shp1358*	23	
**Locus sCDS_273/Ster_RS07530**										
STER_sCDS_273		NR	3	8	1434537	1434608	−	*shp_273_*	23	
STER_RS07530	STER_1530	NR	648	628	1434695	1435558	+	*rgg_7530_*	287	MutR family transcriptional regulator
STER_RS07535	STER_1531	28.9	19052	660	1435675	1436853	+		392	MFS transporter
**Gene STER_sCDS_560**										
STER_sCDS_560		3.2	39	12	1441243	1441359	+		38	
***pptAB***										
STER_RS07725	STER_1571	37.9	5837	154	1472993	1474030	−	*pptB*	345	ABC transporter permease
STER_RS07730	STER_1572	2491	3491	1	1474027	1474758	−	*pptA*	243	ABC transporter ATP-binding protein
**Locus sCDS_279/Ster_RS09420**										
STER_sCDS_279		1.9	139	74	1785690	1785761	−	*shp_279_*	23	
STER_RS09420		NR	1102	1194	1786014	1786712	+	*rgg_9420_*	232	MutR family transcriptional regulator
STER_sCDS_584		NR	71	92	1786790	1786897	+		35	
STER_sCDS_809		NR	0	0	1786854	1786919	+		21	
STER_sCDS_462		13.1	1242	95	1786909	1787088	+		59	
STER_RS09425	STER_1924	14.9	15002	1007	1787097	1788152	+		351	radical SAM protein
STER_RS09430	STER_1925	15.4	18315	1192	1788145	1789686	+		513	ABC transporter ATP-binding protein
STER_sCDS_680		19.6	150	8	1789819	1789905	+		28	

Only genes with an adjusted *p*-value ≤ 0.01 and an at least 1.9-fold change were examined; NR, not significantly regulated (adjusted *p*-value > 0.01); ND, not determined; * Average of normalized read counts from the three biological replicates.

## Supplementary Materials

The following are available online at https://www.mdpi.com/2073-4425/11/9/1096/s1, Table S1: Oligonucleotides used in this study, Table S2: Plasmids used in this study, Table S3: List of CDSs annotated in GenBank and short putative CDSs predicted by BactgeneSHOW in the chromosome and the two plasmids of the *S. thermophilus* wild-type strain (LMD-9), Table S4: List of the SHP*1358 (MGKKQILLTLLLVVFEGIIVIVVG) fragments whose masses was looked for in the TIL1213 strain (ΔamiCDE pBV5030::P_32_-*shp_1358_*) via LC-MS/MS., Table S5: Genes significantly upregulated in the TIL1560 strain (*comR*::*aphA3*) versus in the TIL1561 strain (*comR*::*aphA3* Δ*eep*) (positively controlled by Eep), Table S6: Genes significantly upregulated in the TIL1560 strain (*comR*::*aphA3*)versus in the TIL1564 strain (*comR*::*aphA3* Δ*amiCDE*) (positively controlled by Ami), Figure S1: Detection of the mature forms of SHP_1299_ and SHP_1358_ in cultures of the *S. thermophilus* wild-type strain (LMD-9) via LC-MS/MS. Fragmentation spectra for SHP_1299_ (A) and SHP_1358_ (B); mass, retention time, and the integrated area under the curve obtained from the extracted-ion chromatograms for both peptides (C). The data shown are representative of three independent experiments, Figure S2: Information on the *shp*/*rgg* loci in the *S. thermophilus* wild-type strain (LMD-9). (A) Schematic representation of the six *shp*/*rgg* loci based on genome annotation for the LMD-9 strain, as described in Table S4. Annotations for the *rgg* genes come from GenBank (CP000419.1), and annotations for the *shp* genes were developed in our lab using BactogeneSHOW, as described in the Materials and methods section. The *rgg* genes and *shp* genes are represented by black and gray arrows, respectively. The size of the protein encoded by each gene is indicated underneath. (B) Alignment of the sequences for the SHP precursors. The predicted or experimentally validated sequences for the mature forms are in bold, Figure S3: Detection of the mature form of SHP_279_ in cultures of the *S. thermophilus* wild-type strain (LMD-9) via LC-MS/MS. Fragmentation spectra for SHP_279_ (A); mass, retention time, and the integrated area under the curve obtained from the extracted-ion chromatograms for the peptide (B). The data shown are representative of three independent experiments.
