# Supplementary material for: Export of Rgg Quorum Sensing Peptides is Mediated by the PptAB ABC Transporter in Streptococcus Thermophilus Strain LMD-9
Source: Genes (Basel). 2020 Sep 19;11(9):1096. doi: 10.3390/genes11091096 (PMC7564271; doi:10.3390/genes11091096)
Supplement: Supplementary file 1 [file genes-11-01096-s001.zip › Supplementary Figures.pptx]

## Slide 1
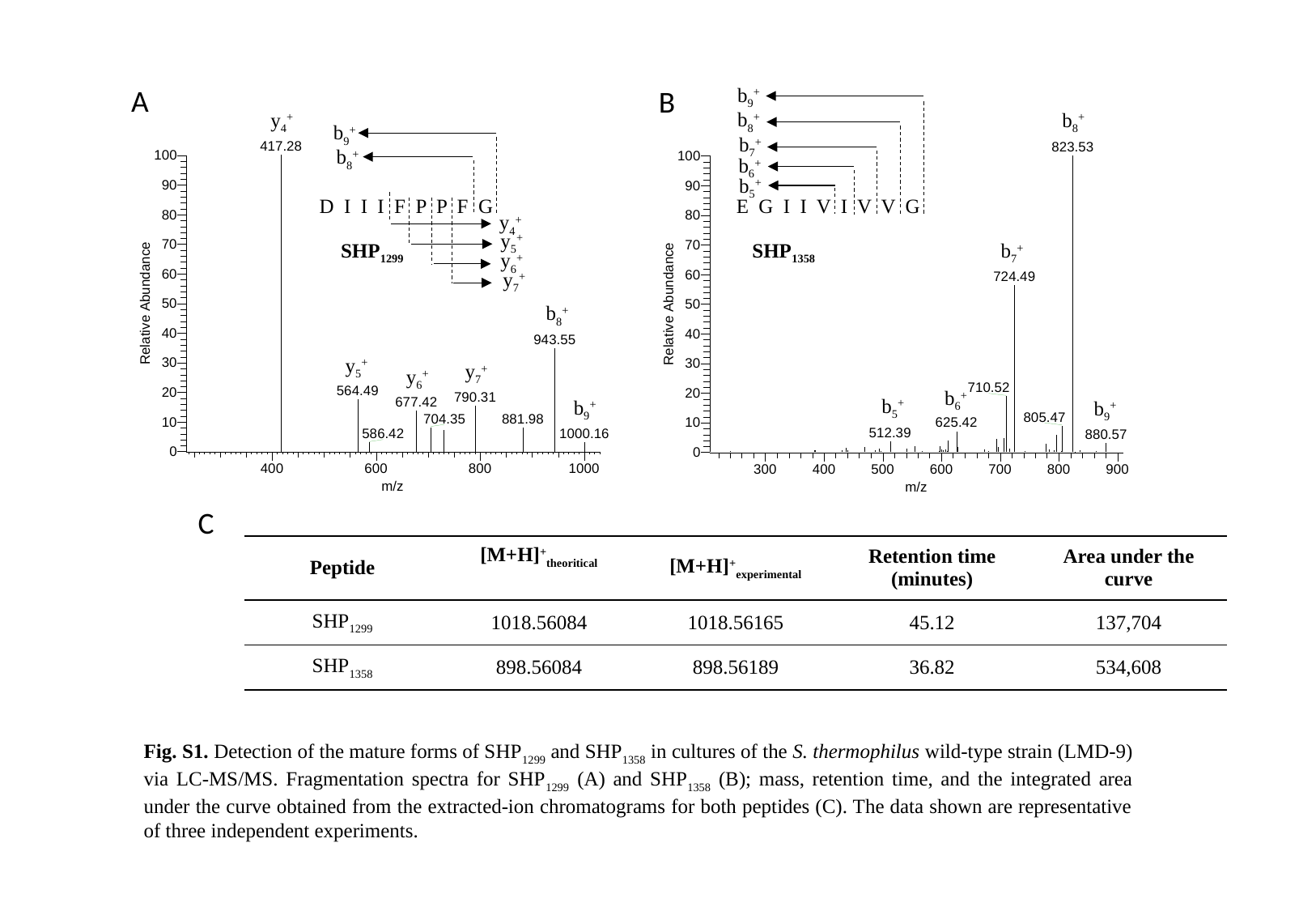

A
b9+
b8+
b8+
b7+
b6+
b5+
b9+
y4+
b8+
y5+
y7+
y6+
b9+
b9+
b7+
b8+
b6+
b5+
D I I I F P P F G
E G I I V I V V G
y4+
y5+
y6+
y7+
B
SHP1299
SHP1358
C
| Peptide | [M+H]+theoritical | [M+H]+experimental | Retention time (minutes) | Area under the curve |
| --- | --- | --- | --- | --- |
| SHP1299 | 1018.56084 | 1018.56165 | 45.12 | 137,704 |
| SHP1358 | 898.56084 | 898.56189 | 36.82 | 534,608 |
Fig. S1. Detection of the mature forms of SHP1299 and SHP1358 in cultures of the S. thermophilus wild-type strain (LMD-9) via LC-MS/MS. Fragmentation spectra for SHP1299 (A) and SHP1358 (B); mass, retention time, and the integrated area under the curve obtained from the extracted-ion chromatograms for both peptides (C). The data shown are representative of three independent experiments.

## Slide 2
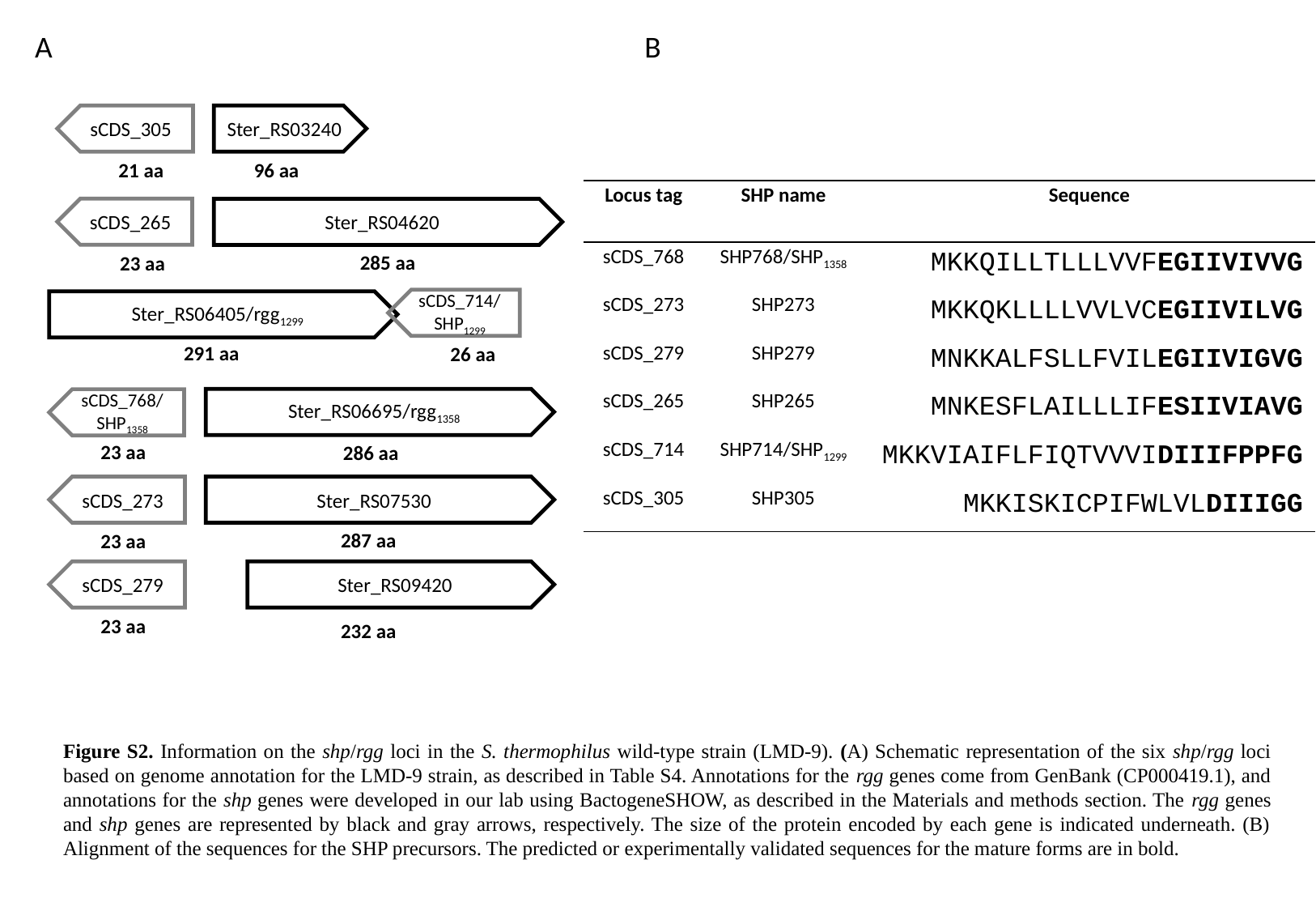

A
B
sCDS_305
Ster_RS03240
96 aa
21 aa
| Locus tag | SHP name | Sequence |
| --- | --- | --- |
| sCDS\_768 | SHP768/SHP1358 | MKKQILLTLLLVVFEGIIVIVVG |
| sCDS\_273 | SHP273 | MKKQKLLLLVVLVCEGIIVILVG |
| sCDS\_279 | SHP279 | MNKKALFSLLFVILEGIIVIGVG |
| sCDS\_265 | SHP265 | MNKESFLAILLLIFESIIVIAVG |
| sCDS\_714 | SHP714/SHP1299 | MKKVIAIFLFIQTVVVIDIIIFPPFG |
| sCDS\_305 | SHP305 | MKKISKICPIFWLVLDIIIGG |
sCDS_265
Ster_RS04620
285 aa
23 aa
sCDS_714/
SHP1299
Ster_RS06405/rgg1299
291 aa
26 aa
Ster_RS06695/rgg1358
sCDS_768/
SHP1358
23 aa
286 aa
sCDS_273
Ster_RS07530
287 aa
23 aa
Ster_RS09420
sCDS_279
23 aa
232 aa
Figure S2. Information on the shp/rgg loci in the S. thermophilus wild-type strain (LMD-9). (A) Schematic representation of the six shp/rgg loci based on genome annotation for the LMD-9 strain, as described in Table S4. Annotations for the rgg genes come from GenBank (CP000419.1), and annotations for the shp genes were developed in our lab using BactogeneSHOW, as described in the Materials and methods section. The rgg genes and shp genes are represented by black and gray arrows, respectively. The size of the protein encoded by each gene is indicated underneath. (B) Alignment of the sequences for the SHP precursors. The predicted or experimentally validated sequences for the mature forms are in bold.

## Slide 3
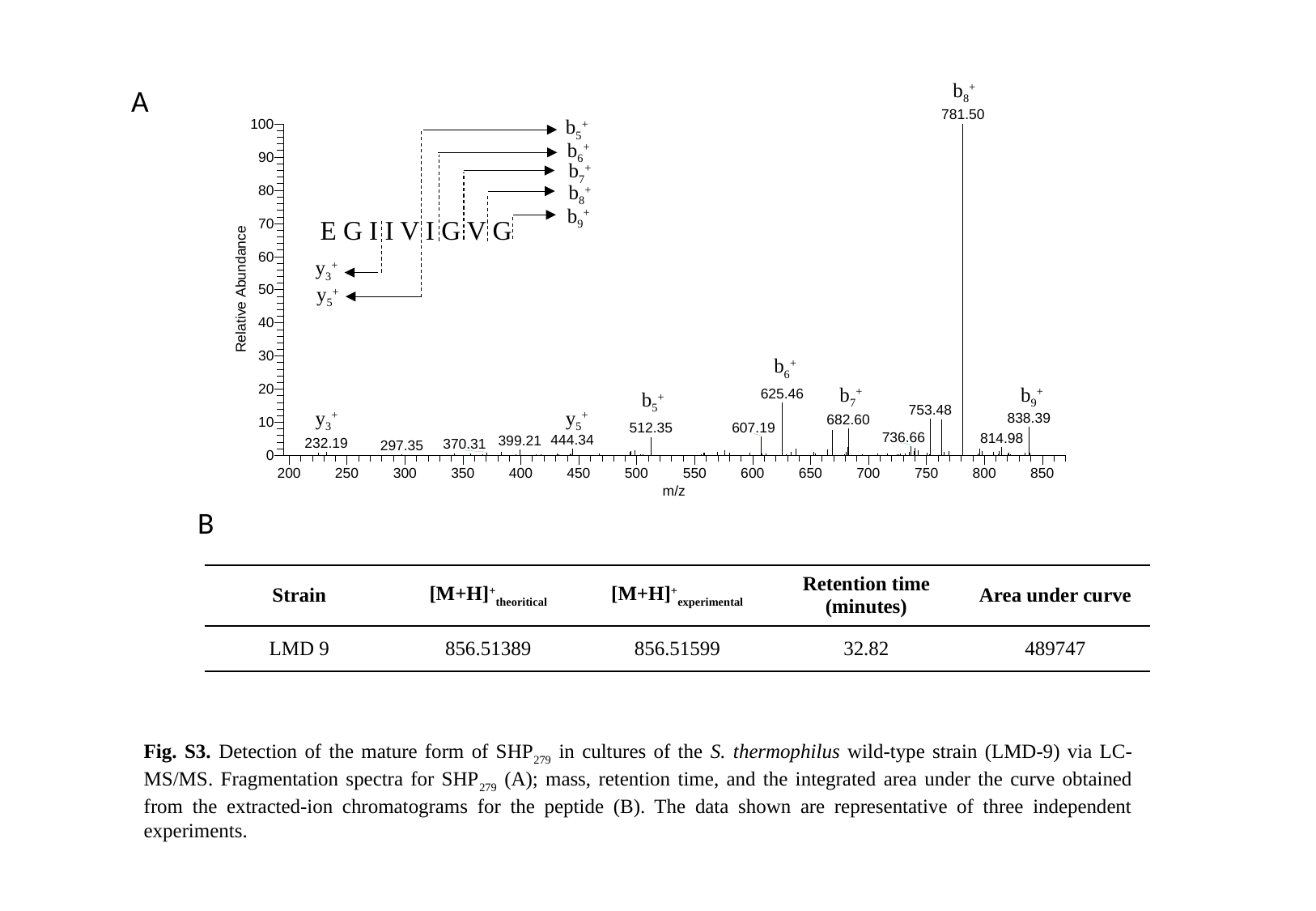

b8+
b6+
b9+
b7+
b5+
y5+
y3+
b5+
b6+
b7+
b8+
b9+
E G I I V I G V G
y3+
y5+
A
B
| Strain | [M+H]+theoritical | [M+H]+experimental | Retention time (minutes) | Area under curve |
| --- | --- | --- | --- | --- |
| LMD 9 | 856.51389 | 856.51599 | 32.82 | 489747 |
Fig. S3. Detection of the mature form of SHP279 in cultures of the S. thermophilus wild-type strain (LMD-9) via LC-MS/MS. Fragmentation spectra for SHP279 (A); mass, retention time, and the integrated area under the curve obtained from the extracted-ion chromatograms for the peptide (B). The data shown are representative of three independent experiments.
