# Supplementary material for: Export of Rgg Quorum Sensing Peptides is Mediated by the PptAB ABC Transporter in Streptococcus Thermophilus Strain LMD-9
Source: Genes (Basel). 2020 Sep 19;11(9):1096. doi: 10.3390/genes11091096 (PMC7564271; doi:10.3390/genes11091096)
Supplement: Supplementary file 1 [file genes-11-01096-s001.zip › Supplementary Tables S1 S2 _VF.docx]

**Table S1**

Oligonucleotides used in this study.

| **Primer** | **Sequence (5’ 🡪 3’)^a,b^** |
| --- | --- |
| Erm-F | GGGACCTCTTTAGCTCCTTGG |
| Erm-R | GGAGATAAGACGGTTCGTGTTCG |
| AphA3-F | ccagcgaaccatttgag |
| AphA3-R | gttgcggatgtacttcag |
| pptABe_up-F | CGAACGTGCTCTTTAGG |
| pptABe_up-R | CTACTGACAGCTTCCAAGGAGCTAAAGAGGTCCCGCTGGATCTTGTTGAATAG |
| pptABe_down-F | GCAAGTCAGCACGAACACGAACCGTCTTATCTCCCTGAAAAATGGCTGGCTATG |
| pptABe_down-R | CATCTTCACAAAGACACG |
| pptAB-SpeI | AACTACTAGTAACACTCATTCCGATAG |
| pptAB-EcoRI | GAAGAATTCAATTTAAGCATTATCTTTTCTCTC |
| pptAB_up-F | GAAGAATTCCAAGAATACAATCCAGG |
| pptAB_up-R | GCATTATCTTTTCTCTCTTTGG |
| pptAB_down-F | TATCATACCAAAGAGAGAAAAGATAATGCCCTGATTTTGATTGGAGTTATG |
| pptAB_down-R | CGGGGTACCTGCCACTTACCTTTAGC |
| comR_up-F | GAAGAATTCTTTTGCTTACAGTTGCC |
| comR _up-R | GACATCTAATCTTTTCTGAAGTACATCCGCAACGTCCAATGCTGTCTTTTAAG |
| comR _down-F | ATAATCTTACCTATCACCTCAAATGGTTCGCTGGTTGGAAGACTCGGTTTTAG |
| comR _down-R | CCGCTCGAGGGATAACAGAAATGGAGAAG |

^a^The recognition sequences for the restriction enzymes are underlined with solid lines.

^b^The inverse and complementary sequences for the erythromycin or kanamycin cassettes are underlined with short dashes, and the inverse and complementary sequence for the *pptAB* upstream fragment is underlined with long dashes.

**Table S2**

Plasmids used in this study.

| **Plasmid** | **Description^a^** | **Source or reference** |
| --- | --- | --- |
| pG^+^host9 | Erm, Ts plasmid | [37] |
| pG^+^host9::updown.*pptAB* | Erm, Ts plasmid used for *pptAB* gene replacement via double crossover integration | This study |
| pG^+^host9::updown.*comRaphA3* | Erm, pG^+^host9 derivative used for *comR* gene replacement with a kanamycin resistance cassette via double crossover integration | This study |
| pGICB004a | Erm,km, pG^+^host9 derivative containing the *luxAB* genes of *Photorhabdus luminescens* and an kanamycin resistance cassette surrounded by two fragments of the *blp* operon, allowing double crossover integration at the *blp* locus of *S. thermophilus* | [33] |
| pGICB004a::P*_pptAB_* | Erm, km, pGICB004a derivative used to introduce a P*_pptAB_*-*luxAB* transcriptional fusion at the *blp* locus of *S. thermophilus* | This study |
| pBV5030::P_32_-*ster_1357* | Erm, replicative plasmid in *E. coli* and Gram-positive bacteria allowing the overexpression of the *ster_1357* gene | [27] |
| pBV5030::P_32_ | Erm, pBV5030::P_32_-*ster_1357* derivative | This study |
| pBV5030::P_32_-*shp_1358_* | Erm, pBV5030::P_32_-*ster_1357* derivative allowing the overexpression of the *shp_1358_* gene | [27] |

^a^Erm and Km = resistance to erythromycin and kanamycin, respectively; Ts = the plasmid encodes a thermosensitive RepA protein.
