# Supplementary material for: Export of Rgg Quorum Sensing Peptides is Mediated by the PptAB ABC Transporter in Streptococcus Thermophilus Strain LMD-9
Source: Genes (Basel). 2020 Sep 19;11(9):1096. doi: 10.3390/genes11091096 (PMC7564271; doi:10.3390/genes11091096)
Supplement: Supplementary file 1 [file genes-11-01096-s001.zip › Supplementary Tables S4_VF.docx]

**Table S4**

List of the SHP*_1358_ (MGKKQILLTLLLVVF**EGIIVIVVG)** fragments whose masses was looked for in the TIL1213 strain (Δ*amiCDE* pBV5030::P_32_-*shp_1358_*) via LC-MS/MS.

| **Annotation** | **Sequence** | **[M+H^+^]^+^** | **[M+2H^+^]^2+^** | **[M+3H^+^]^3+^** |
| --- | --- | --- | --- | --- |
| SHP*_1358_^1-15^ | MGKKQILLTLLLVVF | 1716.08564 | 858.54648 | 572.70009 |
| SHP*_1358_^2-15^ | GKKQILLTLLLVVF | 1585.04515 | 793.02624 | 529.01993 |
| SHP*_1358_^3-15^ | KKQILLTLLLVVF | 1528.02369 | 764.51551 | 510.01278 |
| SHP*_1358_^4-15^ | KQILLTLLLVVF | 1399.92873 | 700.46802 | 467.31446 |
| SHP*_1358_^5-15^ | QILLTLLLVVF | 1271.83376 | 636.42054 | 424.61614 |
| SHP*_1358_^6-15^ | ILLTLLLVVF | 1143.77519 | 572.39125 | 381.92994 |
| SHP*_1358_^7-15^ | LLTLLLVVF | 1030.69112 | 515.84922 |  |
| SHP*_1358_^1-14^ | MGKKQILLTLLLVV | 1569.01722 | 785.01227 | 523.67729 |
| SHP*_1358_^1-13^ | MGKKQILLTLLLV | 14469.94881 | 735.47807 | 490.65448 |
| SHP*_1358_^1-12^ | MGKKQILLTLLL | 1370.88040 | 685.94386 | 457.63168 |
| SHP*_1358_^1-11^ | MGKKQILLTLL | 1257.79633 | 629.40183 | 419.93699 |
| SHP*_1358_^1-10^ | MGKKQILLTL | 1144.71227 | 572.85980 |  |

SHP*_1358_ is a 24-amino-acid-long peptide containing a glycine residue between the methionine at position 1 and the lysine at position 2 in the wild-type sequence for SHP_1358_. The sequence of the active mature peptide is in bold.
